# Dose-dependent regulation of kidney mitochondrial function by angiotensin II

**DOI:** 10.48101/ujms.v128.10312

**Published:** 2023-12-21

**Authors:** Ebba Sivertsson, Amanda Balboa, Tomas A Schiffer, Peter Hansell, Malou Friederich-Persson, Patrik Persson, Fredrik Palm

**Affiliations:** a Department of Medical Cell Biology, Uppsala University, Uppsala, Sweden; b Department of Physiology and Pharmacology, Karolinska Institute, Stockholm, Sweden

**Keywords:** Angiotensin II, mitochondria function, mitochondria leak respiration, uncoupling, oxidative stress, kidney, renin-angiotensin II-aldosterone system

## Abstract

**Background:**

Intrarenal hypoxia has been suggested a unifying pathway to chronic kidney disease (CKD) and increased mitochondria leak respiration, which increases mitochondrial oxygen usage and is one important mechanism contributing to the development of the hypoxia. Previous studies indicate that angiotensin II (Ang II) effects on mitochondria function could be dose dependent. We investigated how moderate and high levels of Ang II affect kidney mitochondria function and pathways of leak respiration.

**Methods:**

C57 black 6 mice were treated with either vehicle or Ang II in low dose (400 ng/kg/min) or high dose (1,000 ng/kg/min) for 4 weeks. The function of kidney cortex mitochondria was measured by high-resolution respirometry. Ang II effects on gene expression in kidney tissue were measured by quantitative real-time PCR. Thiobarbituric acids reactive substances were determined as a marker of oxidative stress, and urinary protein excretion was measured as a maker of kidney injury.

**Results:**

Low-dose Ang II induced overall mitochondria respiration, without compromising capacity of ATP production. Mitochondrial leak respiration was increased, and levels of oxidative stress were unchanged. However, high-dose Ang II decreased overall mitochondria respiration and reduced mitochondrial capacity for ATP production. Mitochondrial leak respiration was decreased, and oxidative stress increased in kidney tissue. Furthermore, gene expression of mediators that stimulate vasoconstriction and ROS production was increased, while components of counteracting pathways were decreased.

**Conclusions:**

In conclusion, Ang II dose-dependently affects mitochondrial function and leak respiration. Thus, Ang II has the potential to directly affect cellular metabolism during conditions of altered Ang II signaling.

## Introduction

Chronic kidney disease (CKD) is a worldwide health problem (1), and the two most common causes are diabetes and hypertension (2). Regardless of initial disorder, intrarenal hypoxia has emerged as a unifying pathway to CKD (3–6). The proposed mechanisms causing tissue hypoxia are many and often overlapping. However, oxidative stress, bioavailability of nitric oxide (NO), and increased mitochondria oxygen consumption have been shown to be involved (5).

The majority of cellular oxygen consumption (QO_2_) is due to mitochondrial respiration in order to sustain cellular energy production. Mitochondria produce adenosine triphosphate (ATP) through the process of oxidative phosphorylation where QO_2_ is coupled to phosphorylation of adenosine diphosphate (ADP) to produce ATP. This oxygen consuming process is dependent on adenine nucleotide translocase (ANT) to supply the ATP synthase with substrate, which is done by exchanging the newly produced ATP for cytosolic ADP. However, the coupling of QO_2_ to ATP production is not absolute, and protons can pass across the mitochondrial membrane via additional mechanisms. This leak of protons across the inner membrane is either via regulated proteins, such as uncoupling proteins (UCP) or ANT, or unregulated diffusion-like processes. Importantly, these alternative proton-leak pathways increase mitochondrial QO_2_ in order to sustain ATP production.

Although ubiquitously expressed, UCP2 has been demonstrated to be the predominant isoform expressed in the healthy kidney (7). By allowing protons to be transported across the membrane into the mitochondrial matrix, UCP2 has the potential to decrease the proton gradient and bypass ATP production. This has been proposed as a defense mechanism to regulate the production of reactive oxygen species (ROS) by the electron transport chain (ETC) in conditions of pathologically high mitochondrial membrane potential (8).

Accumulating evidence indicates that UCP2 has a central role in the development of diabetic kidney disease, causing mitochondria dysfunction and associated increase in total kidney QO_2_ and development of kidney tissue hypoxia (9, 10). Furthermore, the involvement of UCP2-mediated mitochondrial leak respiration in diabetic nephropathy has been demonstrated in UCP2 deficient (UCP2^-/-^) diabetic mice, in which the absence of UCP2 protected from diabetic-induced injury (11).

Additional to ADP/ATP transport, in diabetes, ANT has been demonstrated to mediate mitochondrial leak respiration and contribute tissue hypoxia, similar to UCP2 (9). Also, acute knock down of UCP2 by siRNA in diabetic rats induced a compensatory increase in ANT-mediated mitochondria leak respiration and reduced oxidative stress (12), indicating a close relationship between UCP2 and ANT in kidney mitochondria. A protein-protein interaction between UCP2 and ANT and a coregulation of the two have recently been proposed by Schiffer et al. (13). They show that ANT-regulated mitochondrial leak respiration was decreased by preceding UCP2 inhibition and, interestingly, preceding ANT inhibition completely abolished UCP2-mediated mitochondrial leak respiration. This provides exciting evidence that UCP2 and ANT activities are coregulated, and that the UCP2 activity is dependent on a functional ANT.

In hypertension, angiotensin II (Ang II) has a central role in the development of endothelial dysfunction and damage by inducing mitochondria dysfunction and increased ROS production (14). In Ang II-induced aortic aneurysm, UCP2 counteracts Ang II-stimulated ROS production, evident by increased incidence of aneurysms and oxidative stress in UCP2^-/-^ mice (15). However, the effect of Ang II on UCP2 and ANT-mediated leak respiration in the kidney is less clear, although UCP2^-/-^ mice develop more profound kidney injury in response to increased Ang II signaling when combined with high salt diet (16). In isolated mitochondria from diabetic animals, Ang II-induced dose-dependent inhibition of mitochondria respiration and leak respiration via UCP2 was affected by Ang II receptor inhibition (17).

The aim of this study was to compare the effects of different plasma levels of Ang II on kidney mitochondria function, leak respiration, and production of ROS in kidney tissue. In order to do so, mice were chronically administered two different doses of Ang II and compared to vehicle-treated controls.

## Materials and methods

### Animals and experimental groups

The study protocol was reviewed and approved by the Swedish Ethical Review Authority (approval number 5.8.18-13970/2018 and date of approval 2018-10-26). This study was carried out in accordance with the NIH Guide for the Care and Use of Laboratory Animals. Animals were housed under controlled conditions with a 12 h light/dark cycle and free access to tap water and standard rodent chow.

Male and female C57 black 6 mice were randomly allocated into one of three treatment groups to receive either vehicle (saline), low-dose (400 ng/kg/min), or high-dose (1,000 ng/kg/min) Ang II, delivered by osmotic minipumps (model 1004, Alzet, Cupertino, CA, USA) for 4 weeks. Minipumps were inserted subcutaneously under Isoflurane anesthesia, and analgesia (Karprofen; 5 mg/kg; Bela-Pharm GmbH & Co, Vechta, Germany) was given perioperative and 24 h post-surgery.

### Metabolic cages and measurements of urinary protein leakage

Before insertion of osmotic minipumps (baseline) and after 4 weeks of chronic treatments, animals were placed in metabolic cages for 24 h to measure water intake and urine production. Body weight was recorded at baseline and at 4 weeks of chronic treatment before animals were put in metabolic cages. Urinary protein concentration was measured by DC Protein Assay (Bio-Rad Laboratories, CA, USA) and multiplied by urine flow to determine urinary excretion rate.

### Mitochondria isolation and in vitro measurements of mitochondria function

After 4 weeks of treatment, animals were anesthetized with Isoflurane and euthanized by cervical dislocation. Kidneys were removed and transferred to ice-cold isolation buffer (250 mM sucrose, 10 mM 4-(2-hydroxyethyl)-1-piperazineethanesulfonic acid (HEPES), 1 mM ethylene glycol tetraacetic acid (EGTA), and 1 g/L bovine serum albumin (BSA), pH 7.4 compensated with KOH). Kidney cortex was dissected on ice, and tissue samples were snap frozen using liquid nitrogen for later analysis. Remaining kidney cortex was weighed and thereafter homogenized on ice in 4 mL isolation buffer. The homogenate was transferred to falcon tubes and centrifuged at 700 × g for 10 min. The supernatant was pipetted into Eppendorf tubes and centrifuged at 10.000 × g for 10 min. The buffy coat was gently removed with isolation buffer, and the pellet resuspended in 450 µL isolation buffer and centrifuged at 7,000 × g for 5 min. After careful washing with isolation buffer, the pellet was dissolved in pre-calculated volume of preservation buffer (0.5 mM EGTA, 3 mM MgCl_2_, 60 mM K-lactobionate, 20 mM taurine, 10 mM KH_2_PO_4_, 20 mM HEPES, 110 mM sucrose, 20 mM histidine, 20 µM vitamin E succinate, 3 mM glutathione, 1 µM leupeptine, 2 mM glutamate, 2 mM malate, 2 mM Mg-ATP, and 1 g/L BSA essentially fatty-acid free; 0.8 µL/mg sample weight).

Function of kidney cortex mitochondria was measured at 37°C using an Oxygraph 2k (Oroboros Instruments, Innsbruck, Austria). Isolated mitochondria were added to the chamber containing air-equilibrated respiration buffer (0.5 mM EGTA, 3 mM MgCl_2_, 60 mM K-lactobionate, 20 mM taurine, 10 mM KH_2_PO_4_, 20 mM HEPES, 110 mM sucrose, and 1 g/L BSA essentially fatty-acid free) and 5 mM pyruvate and 2 mM malate as substrates for complex I. Baseline respiration (State 4) was determined in the absence of ADP. Maximal respiration of complex I (State 3) was determined by adding 2.5 mM ADP. The degree of coupling of oxygen consumption to ATP production, that is, the respiratory control ratio (RCR), was calculated State 3/State 4. A 10 mM succinate was added to obtain the maximal respiration of both complexes I and II. Thereafter, mitochondria were left to respire to anoxia, and the mitochondrial oxygen affinity (p50) was estimated by determining the oxygen tension, where mitochondria respiration was 50% of maximal respiration. DatLab2 software (Oroboros, Austria) was used for the analysis of p50_mito_ for oxygen, including signal distortion dependent on the delay in response time of the oxygen sensor and correction for internal zero drift of the oxygen sensor, as reported earlier (18). The range of oxygen tension for analysis was set to <2.5 kPa, to fully cover the hyperbolic function of respiration.

Proton-leak-mediated respiration was estimated in a separate chamber containing respiration buffer, 5 mM pyruvate, and 2 mM malate. To measure protein function of key mediators of proton leak, mitochondria was incubated with, in sequence, 2.5 µM oligomycin to inhibit ATP-synthase; 2 mM guanosine diphosphate (GDP) to inhibit UCP-dependent respiration, and 5 µM carboxyatractyloside (CAT) to inhibit ANT-dependent respiration. Protein concentration of the mitochondria suspension was determined spectrophotometrically (DC Protein Assay, Bio-Rad, Hercules, CA, USA) to normalize all measurements for protein content.

### Analysis of gene expression by qPCR

Total RNA from kidney tissue was extracted with Trizol (Invitrogen; Calsbad, CA; USA), according to manufacturer’s instructions, followed by DNase I treatment (Thermo Fisher Scientific; Austin, TX, USA). Conversion to cDNA was performed using the High Capacity cDNA Reverse Transcription Kit (Applied Biosystems; Foster City, CA, USA). Amplification and detection of genes of interest was achieved with QuantStudio5 Real-Time PCR System (Applied Biosystems) by using SYBR Green PCR Master Mix (Applied Biosystems) for fluorescence detection and primers as listed in Table 1. The expression was normalized to the house keeping gene GAPDH. Relative expression of target genes to control (vehicle) was calculated using the 2^-∆∆Ct^ method.

**Table 1:** Forward and reverse primers used in the amplification of the genes of interest.

Gene	Primer foreward	Primer reverse
SOD1	GAGACCTGGGCAATGTGACT	TTGTTTCTCATGGACCACCA
SOD2	GGCCAAGGGAGATGTTACAA	GCTTGATAGCCTCCAGCAAC
SOD3	CTGCTGCTCGCTCACATAAC	TGCTAGGTCGAAGCTGGACT
Cat	ACATGGTCTGGGACTTCTGG	CAAGTTTTTGATGCCCTGGT
ACE1	AGCCACTGACAGAATGGCTC	TGCGCGAGCGGTGTTT
ACE2	TCTGGGAATGAGGACACGGA	CCATAGGCATGGGATCGTGG
AT1aR	ATCGCAGCGGTCTCCTTTT	CGTGGGTCTCCATTGCTAATG
AT1bR	CTCTTTCCTACCGCCCTTCA	TGGCTTCTACTGTCAGGGGAT
AT2R	TGATGCCTTCTTGGGGGTAA	GGAACTGTGCCCAGAAATGC
Mas1	CTGAGTTTGGAAGCCTCTGG	TTCCTTAAACATGCCCGTTC

### Analysis of TBARS

Thiobarbituric acids reactive substances (TBARS) in kidney cortex were determined fluorometrically. Tissue samples were homogenized in ice cold distilled water. Malondialdehyde was used to prepare standard samples. A 50 μL sample was mixed with 42 μL 0.67% thiobarbituric acid and heated to 97°C for 60 min. After cooling on ice, 50 μL methanol:1 mM NaOH (91:9) was added, the samples vortexed and centrifuged at 3,000 rpm for 5 min at room temperature. The supernatant was transferred to a 384 well plate, and fluorescence was measured at excitation/emission of 532/553 nm. All values were corrected for protein concentration.

### Statistical analysis

When comparing data obtained at baseline and at 4 weeks within each group, Student t-test (paired and two-tailed) was used. Data between groups were analyzed using a one-way analysis of variance (ANOVA). ANOVA was followed by Tukey’s Multiple Comparison *post hoc* test, when appropriate. All data are presented as means ± Standard Error of the Mean (SEM). *P*-value < 0.05 was considered statistically significant.

## Results

### Data from metabolic cages and proteinuria

There was no difference in 24 h water intake and 24 h urine production or proteinuria at baseline between groups. Animals treated with high-dose Ang II had an increased 24 h water intake and 24 h urine production after 4 weeks of treatment compared to baseline values and compared to other groups at 4 weeks of treatment. There was no difference in weight gain in animals between groups during the treatment period (Table 2).

**Table 2:** Weight gain, 24 h water intake, and 24 h urinary production in mice receiving chronic treatment with vehicle, low-dose (400 ng/kg/min), or high-dose (1,000 ng/kg/min) angiotensin II for 4 weeks.

Treatment	*n*	Water (mL/24 h) Baseline	4 weeks	Urine flow(µL/min) Baseline	4 week	Proteinuria(µg/min) Baseline	4 week	Weight gain (g) 4 weeks
Vehicle	8	1.9 ± 0.3	2.5 ± 1.1	0.6 ± 0.1	0.7 ± 0.1	12.5 ± 2.3	11.4 ± 1.4	-0.3 ± 0.3
Low-dose angiotensin II	6–7	1.8 ± 0.5	2.8 ± 0.9	0.6 ± 0.2	0.6 ± 0.1	10.2 ± 4.4	12.9 ± 4.3	0.6 ± 0.2
High-dose angiotensin II	7–8	1.9 ± 0.5	4.2 ± 0.6*	0.7 ± 0.1	1.5 ± 0.2*^#^	12.1 ± 2.0	11.2 ± 1.7	-0.3 ± 0.5

All values are means ± SEM.

* denotes *P* < 0.05 compared to vehicle-treated group, and

# denotes *P* < 0.05 compared to low-dose treatment.

### Respiration of isolated mitochondria

Treatment with low-dose Ang II increased overall mitochondria respiration, both in the absence of ADP (State 4) and after addition of ADP to stimulate maximal oxidative phosphorylation (State 3). High-dose Ang II decreased overall mitochondria respiration compared to control. There was no difference in oxygen affinity of mitochondria between groups (Table 3).

**Table 3:** Analysis of maximal oxidative capacity and oxygen affinity in isolated mitochondria from mice receiving chronic treatment with vehicle, low-dose (400 ng/kg/min), or high-dose (1,000 ng/kg/min) angiotensin II for 4 weeks.

Treatment	*n*	State 4 respiration (pmol O_2_/s/mg protein)	Complex I dependent State 3 respiration (pmol O_2_/s/mg protein)	Complex I+II dependent State 3 respiration (pmol O_2_/s/mg protein)	P50 (kPa)
Vehicle	7–8	0.78 ± 0.03	5.8 ± 0.3	13.5 ± 0.3	0.083 ± 0.004
Low-dose angiotensin II	6–8	1.06 ± 0.06*	8.5 ± 0.7*	16.1 ± 1.2	0.075 ± 0.002
High-dose angiotensin II	7–8	0.61 ± 0.03*#	3.8 ± 0.3*#	9.0 ± 0.6*^#^	0.086 ± 0.005

P50: mitochondrial oxygen affinity.

All values are means ± SEM.

* denotes *P* < 0.05 compared to vehicle-treated group, and

# denotes *P* < 0.05 compared to low-dose treatment.

**Figure 1:**
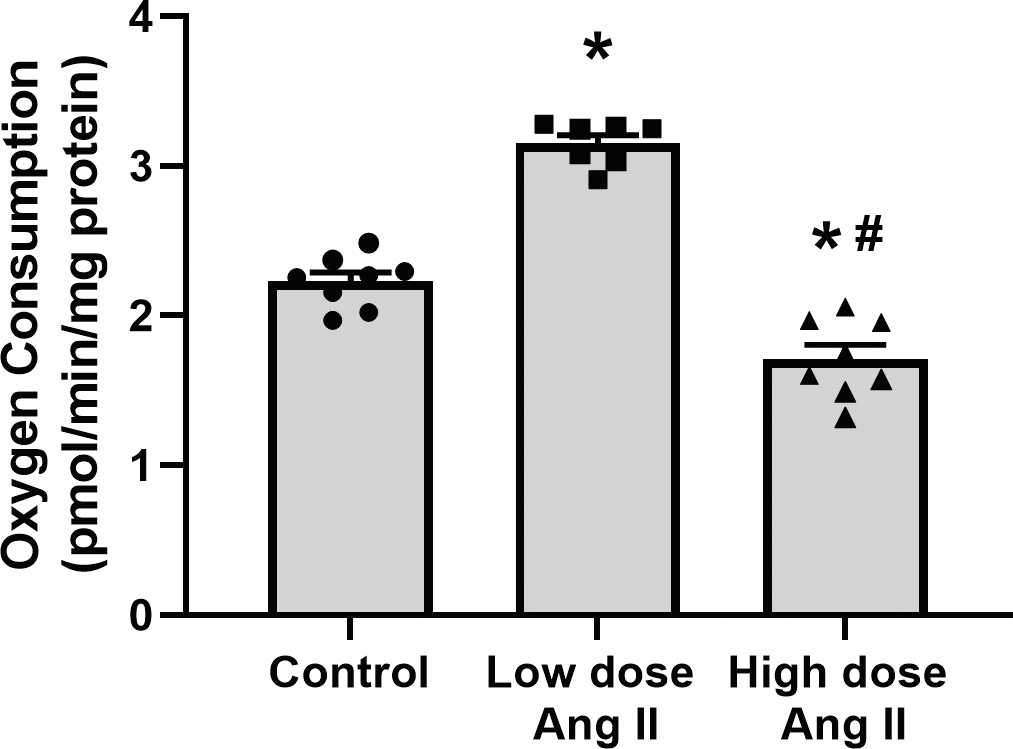
Estimation of unregulated leak respiration of isolated kidney cortex mitochondria of C57 black 6 mice receiving vehicle, low-dose (400 ng/kg/min) angiotensin II (Ang II), or high-dose (1,000 ng/kg/min) Ang II for 4 weeks. Panels show absolute oxygen consumption (QO_2_) after the addition of oligomycin to inhibit the ATP synthase and QO_2_ related to ATP production. Data presented as means ± SEM. * denotes *P* < 0.05 compared to vehicle-treated group, and # denotes *P* < 0.05 compared to low-dose treatment.

**Figure 2:**
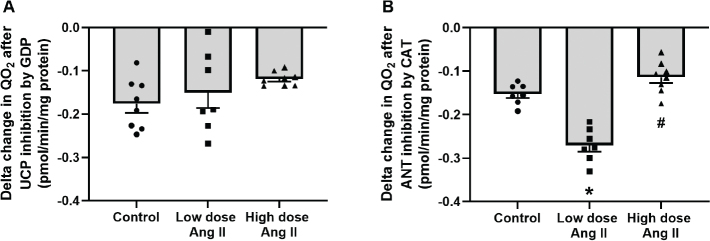
Pathways of regulated leak respiration of isolated kidney cortex mitochondria of C57 black 6 mice receiving vehicle, low-dose (400 ng/kg/min) angiotensin II (Ang II), or high-dose (1,000 ng/kg/min) Ang II for 4 weeks. Panels show delta change in oxygen consumption (QO_2_) after the addition of (A) GDP to inhibit UCP-mediated leak respiration; and (B) CAT to inhibit ANT-mediated leak respiration. Data presented as means ± SEM. * denotes *P* < 0.05 compared to vehicle-treated group, and # denotes *P* < 0.05 compared to low-dose treatment.

**Figure 3:**
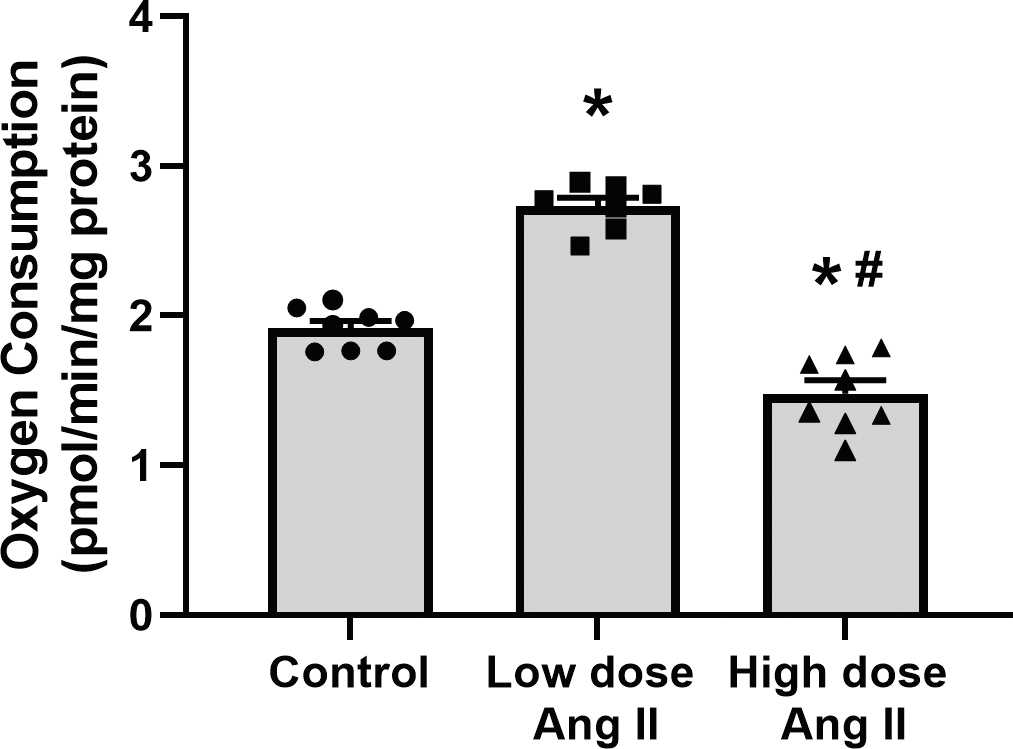
Estimation of unregulated leak respiration of isolated kidney cortex mitochondria of C57 black 6 mice receiving vehicle, low-dose (400 ng/kg/min) angiotensin II (Ang II), or high-dose (1,000 ng/kg/min) Ang II for 4 weeks. Panels show absolute oxygen consumption (QO_2_) after the addition of oligomycin, GDP, and CAT to inhibit regulated pathways of QO_2_. Data presented as means ± SEM. Statistical comparisons between all groups were made using a one-way ANOVA followed by Tukey’s post hoc test. * denotes *P* < 0.05 compared to vehicle-treated group, and # denotes *P* < 0.05 compared to low-dose treatment.

Total leak respiration was increased by low-dose Ang II and decreased by high-dose Ang II (Figure 1). Incubation with GDP to block UCP-mediated leak respiration had no effect in either group (Figure 2a). However, the addition of CAT to block ANT-mediated leak respiration had an increased effect in low-dose-treated animals compared to control, indicating an increased activity of ANT in this group. This effect was significantly decreased in the high-dose-treated group (Figure 2b). Respiration after incubation with oligomycin, GDP, and CAT to block QO_2_ related to ATP production and pathways of regulated leak is an estimate of unregulated leak, that is, basal leak of protons over the mitochondrial membrane. Animals treated with low-dose Ang II had increased unregulated leak respiration compared to controls, whereas it was decreased in the high-dose-treated group (Figure 3).

**Figure 4:**
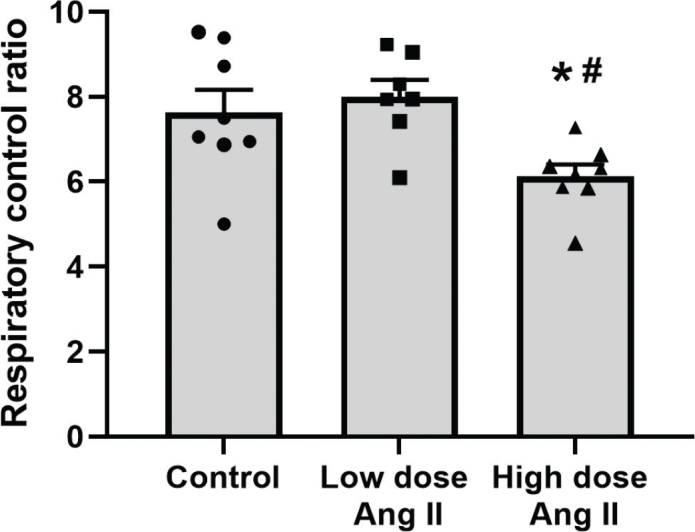
Respiratory control ratio of isolated kidney cortex mitochondria of C57 black 6 mice receiving vehicle, low-dose (400 ng/kg/min) angiotensin II (Ang II), or high-dose (1,000 ng/kg/min) Ang II for 4 weeks. Data presented as means ± SEM. * denotes *P* < 0.05 compared to vehicle-treated group, and # denotes *P* < 0.05 compared to low-dose treatment.

The capacity of ATP production, that is, the RCR, was unchanged in low-dose-treated animals but reduced in high-dose group compared to controls (Figure 4).

### Gene expression

Gene expression of important mediators of antioxidant defense systems, that is, superoxide dismutase (SOD) 1 and 2, and catalase, was increased by low-dose Ang II compared to vehicle-treated controls. SOD2 expression was also increased by high-dose treatment, but SOD1 and catalase were unchanged compared to control group. The expression of SOD3 was unaffected in both groups (Table 4).

**Table 4:** Gene expression of antioxidant defense systems in kidney tissue from mice receiving chronic treatment with vehicle, low-dose (400 ng/kg/min), or high-dose (1,000 ng/kg/min) angiotensin II for 4 weeks.

Treatment	N	SOD1	SOD2	SOD3	Cat
Vehicle	5–7	1.0 ± 0.2	1.0 ± 0.4	1.0 ± 0.1	1.0 ± 0.3
Low-dose angiotensin II	6–7	1.7 ± 0.1*	3.1 ± 0.2*	1.3 ± 0.2	2.7 ± 0.3*
High-dose angiotensin II	5–6	0.8 ± 0.1#	3.7 ± 0.4*	1.3 ± 0.1	1.8 ± 0.3

SOD: superoxide dismutase; Cat: catalase.

Data are presented as fold increase in gene expression compared to vehicle-treated control group (2^-∆∆Ct^). All values are means ± SEM.

* denotes *P* < 0.05 compared to vehicle-treated group, and

# denotes *P* < 0.05 compared to low-dose treatment.

Low-dose Ang II treatment induced a 2-fold increase in the expression of Ang II type 1a receptor (AT1aR), concomitantly with a 2- and 2.4-fold increase of Mas1 receptor and ACE2, respectively. Other important mediators of the renin–angiotensin II–aldosterone system (RAAS) were unaltered. High-dose Ang II induced a 4-fold increase in AT1aR expression and decreased AT2R compared to control group. Furthermore, the expression of angiotensin converting enzyme (ACE) 1 and 2 was decreased compared to low-dose treatment. AT1bR expression was unchanged in both treatment groups (Table 5).

**Table 5:** Gene expression of key enzymes and receptors of the renin-angiotensin-aldosterone system, in kidney cortex tissue from mice receiving chronic treatment with vehicle, low-dose (400 ng/kg/min), or high-dose (1,000 ng/kg/min) angiotensin II for 4 weeks.

Treatment	*n*	ACE1	ACE2	AT1aR	AT1bR	AT2R	Mas1
Vehicle	5–7	1.0 ± 0.1	1.0 ± 0.2	1.0 ± 0.1	1.0 ± 0.2	1.0 ± 0.2	1.0 ± 0.1
Low-dose angiotensin II	6–7	1.0 ± 0.0	2.4 ± 0.5*	2.0 ± 0.2*	1.5 ± 0.3	0.9 ± 0.2	2.0 ± 0.5*
High-dose angiotensin II	6	0.6 ± 0.1*^#^	0.8 ± 0.1^#^	4.3 ± 0.5*^#^	1.3 ± 0.2	0.5 ± 0.0*	1.2 ± 0.3

ACE: angiotensin converting enzyme; AT1aR: angiotensin type 1a receptor; AT1bR: angiotensin type 1b receptor; AT2R: angiotensin type 2 receptor.

Data are presented as fold increase in gene expression compared to vehicle-treated control group (2^-∆∆Ct^). All values are means ± SEM.

* denotes *P* < 0.05 compared to vehicle-treated group, and

# denotes *P* < 0.05 compared to low-dose treatment.

### Oxidative stress status

**Figure 5:**
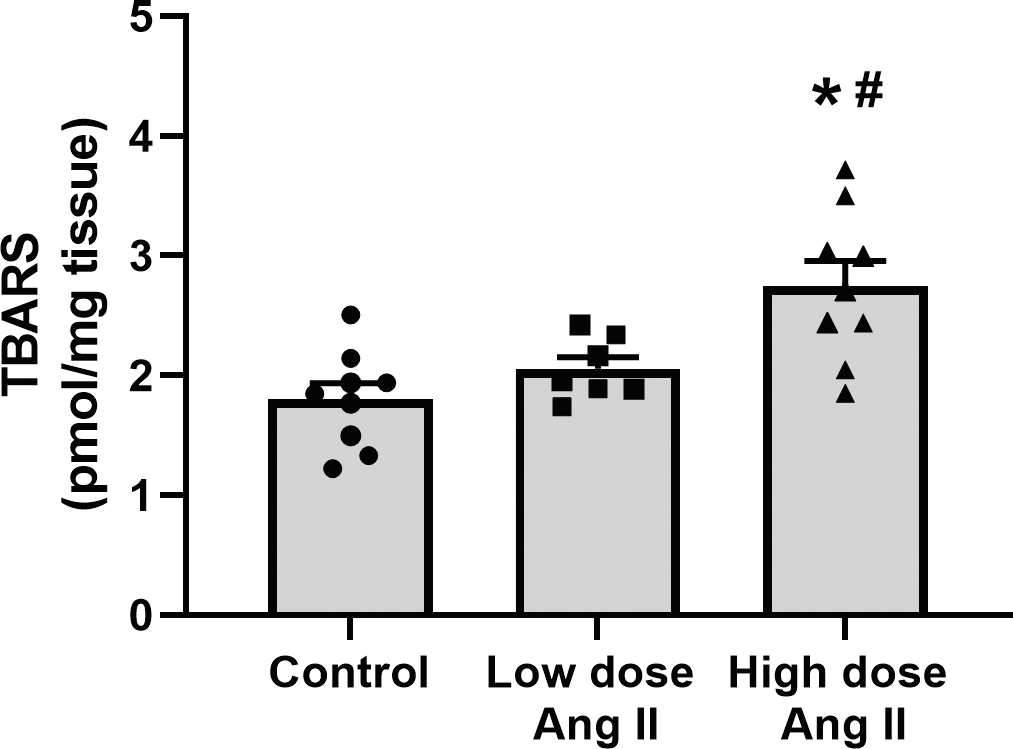
Thiobarbituric acids reactive substances (TBARS) in kidney cortex tissue from C57 black 6 mice receiving vehicle, low-dose (400 ng/kg/min) angiotensin II (Ang II), or high-dose (1,000 ng/kg/min) Ang II for 4 weeks. Values are presented as means ± SEM. * denotes *P* < 0.05 compared to vehicle-treated group, and # denotes *P* < 0.05 compared to low-dose treatment.

TBARS, a marker of oxidative stress status, increased in response to high-dose Ang II compared to controls but unaffected by low-dose treatment (Figure 5).

## Discussion

The main finding of this study is that the effect of Ang II on mitochondrial function is dose dependent. Low-dose Ang II (400 ng/min/kg) administered to moderately increase circulating levels of Ang II induced an overall increase in mitochondria respiration without compromising ATP production capacity. Regulated leak respiration was increased to efficiently control excessive production of ROS. On the contrary, high-dose Ang II (1,000 ng/min/kg) administered to severely increase circulating levels of Ang II decreased overall mitochondrial respiration, reduced the capacity to produce ATP, and induced oxidative stress in kidney tissue. This supports the role of Ang II as a potent physiological regulator of mitochondria respiration and cellular metabolism in the kidney during conditions of altered Ang II signaling (e.g. hypertension and diabetes).

Ang II is mostly known for its central role in the systemic RAAS as a powerful stimulus to increase systemic blood pressure, by increasing vascular tone and fluid reabsorption. Ang II binding to Ang II receptor type 1 (AT1R) mediates the vasoconstriction properties of Ang II. However, the actions of Ang II are not restricted to only vasoconstriction. Signaling via AT2R is a potent pathway to balance the effects of AT1R, stimulating release of NO and inhibiting further RAAS activation. ACE1 cleaves Ang I into the vasoactive form Ang II. To balance the ACE1/Ang II/AT1R pathway, Ang I and Ang II can be further processed by ACE2 into Ang (1–7). By binding to its receptor, Mas1, Ang (1–7) counteracts the effects of Ang II, stimulating NO production and vasodilation. Interestingly, recent evidence of an intracellular RAAS has been demonstrated, with receptors for Ang II expressed in the mitochondria (19).

Due to technical difficulties, we could not obtain blood pressure recordings by tail cuff due to vasoconstriction of the tail vessels, which constitutes a limitation of our study. However, it has been previously demonstrated that 400 and 1,000 ng/min/kg of Ang II infused by osmotic mini pumps induce a dose-dependent increase in blood pressure in the C57 black 6 mouse (20), supporting our assumption that the doses used in this study induce distinctively different levels of Ang II signaling.

Mitochondria from low-dose Ang II-treated animals had increased State 4 respiration and increased respiration during oligomycin incubation, both indicators of increased leak respiration. This could potentially decrease ATP production efficiency; however, RCR was comparable to vehicle-treated controls, indicating that the degree of coupling between QO_2_ and ATP production is unaffected in this group. Although moderately increased Ang II stimulated mitochondrial respiration without compromising the efficiency of ATP production, increased mitochondrial QO_2_ has previously been demonstrated as a pathway to the development of intrarenal hypoxia (9, 21, 22). Intrarenal hypoxia is a common denominator in pathological conditions leading to CKD and has been demonstrated in experimental models of diabetes (23–30), hypertension (31, 32), and CKD (33), as well as in patients with both diabetic (34, 35) and non-diabetic CKD (35). Thus, this increased QO_2_ by mitochondria could be one pathway contributing to the slow, long-term development of CKD in patients with hypertension. These results warrant further studies to investigate the *in vivo* effects on kidney oxygenation and long-term development of CKD.

However, high-dose treatment and severely elevated levels of Ang II induced mitochondria dysfunction, evident by decreased overall mitochondria respiration and reduced RCR. Indeed, it has previously been reported that high concentration of Ang II inhibits mitochondrial QO_2_, an effect directly involving Ang II receptors signaling (17). In isolated mice osteoblasts, Ang II inhibited all respiratory enzymes in the ETC, dissipated mitochondria membrane potential, and decreased mitochondrial ATP production (36). A direct inhibitory effect of Ang II on the mitochondria is likely also in our study. However, high-dose Ang II also reduced ANT activity in isolated mitochondria. Loss of ANT activity could become rate limiting in providing the ATP synthase with the substrate, that is, ADP, for ATP production, which could contribute to decreased mitochondrial efficiency in these animals. Decreased leak respiration would also increase oxidative stress, which is in line with our results demonstrating increased TBARS in kidney cortex of high-dose Ang II-treated animals.

Although UCP2 activity is known to be induced by oxidative stress (37, 38), Ang II treatment did not induce increased uncoupling activity via UCPs susceptible to GDP inhibition in the present study. This is somewhat surprising since Ang II is a powerful stimulator of oxidative stress, and this should, theoretically, induce UCP2-mediated proton leak. However, in previous studies, it has been shown that the addition of an AT2R inhibitor increased UCP leak respiration in isolated mitochondria from diabetic animals (17). Also, studies in spontaneously hypertensive rats showed that AT1R inhibition by losartan increased UCP2 protein content, which successfully reduced mitochondrial production of H_2_O_2_ (39). These results indicate that Ang II has the potential to alter UCP2 protein expression and activity.

Interestingly, uncoupling via ANT is markedly increased by moderately increased Ang II, another effective uncoupler of the mitochondrial membrane. Potentially, ANT could be the main leak pathway in Ang II-induced hypertension, as opposed to UCP-mediated leak that is central in diabetes. This could be either a direct stimulatory effect of Ang II on ANT activity or a compensatory effect to the lack of UCP activity. However, a compensatory increase in ANT activity after the inhibition of UCP2 using small interfering RNA has been previously demonstrated in diabetes, which resulted in increased mitochondrial leak respiration, decreased mitochondria membrane potential, and decreased oxidative stress (12). Furthermore, respiration during incubation with oligomycin + GDP + CAT is increased by low-dose Ang II and decreased by high-dose Ang II. This indicates that Ang II also interacts with the unregulated leak of protons over the mitochondrial membrane, possibly by interacting with the structure of the lipid membrane. Taken together, Ang II has the potential to affect mitochondrial function and respiration at multiple locations of the ETC.

Analyzing gene expression of important mediators of Ang II signaling also revealed a difference between low- and high-dose Ang II treatment. Low-dose Ang II increased the expression of AT1R, but concomitantly also the expression of ACE2 and Mas1. Previous studies from Dynnik *et al.* have demonstrated that low levels of L-arginine or NO donors to isolated liver mitochondria stimulated respiration, whereas high Ang II levels inhibited respiration (40). Increased signaling through the ACE2/Ang (1–7)/Mas1 pathway, stimulating NO release, and balancing the ACE1/Ang II/AT1R pathway would be well in line with our results demonstrating increased mitochondria respiration but unaltered levels of oxidative stress, in animals treated with low-dose Ang II.

High-dose Ang II treatment induced the expression of ACE1, ACE2, and Mas1. Interestingly, the relationship between the two angiotensin receptors was also altered, considerably increasing AT1R expression, while the AT2R expression was decreased. AT1R signaling is associated with the activation of the NADPH oxidase and subsequent increased production of superoxide radicals (14, 41). Indeed, kidney tissue TBARS, a marker of oxidative stress status, was significantly elevated in this treatment group. This was accompanied by decreased gene expressions of important antioxidant defense systems such as SOD2 and catalase. Also, leak respiration via ANT, a powerful resource to counteract ROS production, was decreased by high-dose Ang II. This results in severe mitochondrial dysfunction and oxidative stress in kidney tissue.

Although we could detect differences on a cellular level between low- and high-dose Ang II treatment, we did not see a difference in urinary protein leakage, a common marker of kidney injury. The C57 black 6 mouse has previously been shown to be highly resistant to diabetes-induced kidney injury and proteinuria compared to other mouse strains (42). Possibly, a treatment period of 4 weeks is too short for this resilient strain to develop manifest kidney injury.

In conclusion, the results of this study demonstrate that Ang II can have substantially different effects on mitochondrial function, depending on signal intensity. Moderately increased Ang II stimulates mitochondrial respiration without compromising oxidative phosphorylation capacity and induces ANT-mediated leak respiration, possibly to counteract oxidative stress. This may be an important contributing mechanism for the commonly observed increased kidney oxygen consumption in the early stage of Ang II-dependent hypertension, and thus, a contributing mechanism for the increased long-term risk of CKD development in hypertension. However, severely increased Ang II inhibits mitochondria respiration, reduces oxidative phosphorylation capacity, and increases oxidative stress, possibly making the tissue even more vulnerable to external stress. In addition to any effects on blood flow and oxygen delivery, this dose-dependent effect on mitochondria function might be of importance when studying development of CKD in conditions of altered angiotensin II signaling (e.g. hypertension or diabetes).

## Data Availability

The data that support the findings of this study are openly available at Figshare (https://figshare.com/) at https://doi.org/10.6084/m9.figshare.21276921 and https://doi.org/10.6084/m9.figshare.23795871.
